# Spillover Effects of a Community-Managed Marine Reserve

**DOI:** 10.1371/journal.pone.0111774

**Published:** 2015-04-30

**Authors:** Isabel Marques da Silva, Nick Hill, Hideyasu Shimadzu, Amadeu M. V. M. Soares, Maria Dornelas

**Affiliations:** 1 Departamento de Zoologia, Universidade do Lúrio, Pemba, Mozambique and Departamento de Biologia, Universidade de Aveiro, Aveiro, Portugal; 2 Conservation Programmes, Zoological Society of London, London, England, United Kingdom; 3 Centre for Biological Diversity and Scottish Oceans Institute, School of Biology, University of St. Andrews, St. Andrews, Scotland, United Kingdom; 4 Departamento de Biologia and CESAM, Universidade de Aveiro, Aveiro, Portugal; Aristotle University of Thessaloniki, GREECE

## Abstract

The value of no-take marine reserves as fisheries-management tools is controversial, particularly in high-poverty areas where human populations depend heavily on fish as a source of protein. Spillover, the net export of adult fish, is one mechanism by which no-take marine reserves may have a positive influence on adjacent fisheries. Spillover can contribute to poverty alleviation, although its effect is modulated by the number of fishermen and fishing intensity. In this study, we quantify the effects of a community-managed marine reserve in a high poverty area of Northern Mozambique. For this purpose, underwater visual censuses of reef fish were undertaken at three different times: 3 years before (2003), at the time of establishment (2006) and 6 years after the marine reserve establishment (2012). The survey locations were chosen inside, outside and on the border of the marine reserve. Benthic cover composition was quantified at the same sites in 2006 and 2012. After the reserve establishment, fish sizes were also estimated. Regression tree models show that the distance from the border and the time after reserve establishment were the variables with the strongest effect on fish abundance. The extent and direction of the spillover depends on trophic group and fish size. Poisson Generalized Linear Models show that, prior to the reserve establishment, the survey sites did not differ but, after 6 years, the abundance of all fish inside the reserve has increased and caused spillover of herbivorous fish. Spillover was detected 1km beyond the limit of the reserve for small herbivorous fishes. Six years after the establishment of a community-managed reserve, the fish assemblages have changed dramatically inside the reserve, and spillover is benefitting fish assemblages outside the reserve.

## Introduction

The world’s oceans are subject to a myriad of threats including overexploitation of species, coastal development, land-based pollution, energy practices, aquaculture, land use and transformation, water use, shipping practices, and climate change [1,2,3]. These threats, coupled with continued growth of the human population and migration to coastal areas, are driving unanticipated, unprecedented and complex changes on the world’s oceans [4]. Marine Protected Areas (MPAs) are one of the most often advocated management options to protect oceans from these threats. They are one of the easiest management approaches for non-specialists to grasp, making MPAs an alluring alternative to complex arrays of management tools [5,6]. MPAs also represent a more holistic approach to management. However, their design is often more political and social than based in ecological and fisheries science [7], and can be implemented in situations of limited information [8]. MPAs are widely used, and their use is likely to increase in the future. For example, several large scale marine reserves, the size of California or bigger, were declared around the world to fulfill the goal of 10% of oceans protected as MPAs by 2020 proposed by the Convention on Biological Diversity. Since 1990, MPAs have increased in number by 58% and in extent by 48% [9].

An MPA can have different zones, including: no-take areas, where fishing is prohibited or restricted (e.g. only some gear types allowed); buffer zones; and zones reserved for different activities like sport fishing or aquaculture [10]. Larger MPAs allow different zoning for different activities, providing spatial separation of incompatible human activities and reducing conflict among stakeholders [11]. The most restrictive MPAs are Marine Reserves (MRs), normally dedicated to the protection of biodiversity and ecosystems. To maximize fisheries benefits, networks of several small MRs tend to work better than fewer, bigger MRs, but if the goal is conservation a smaller number of larger MRs is better [12].

MPAs can be established with different goals. They are a central tool for ecosystem-based management, conferring protection on species and habitats from fishing within their borders and also issuing control measures for pollution, gas and oil exploration, and coastal development [13,14,15]. They are implemented for biodiversity conservation and to protect certain zones for underwater tourism, which has become very important for many island and coastal countries [16,17]. However, the most common, and most controversial, goal of MPAs is to enhance fisheries [8,18,19,20,21,22,23,24] through the export of larvae and adults from the protected areas into the surrounding unprotected areas [25]. Despite the potential benefits of MPAs, prohibiting extractive uses can have socio-economic costs such as the loss of income from fishing, and/or the increased costs of having to fish further away [26, 27]. It can be difficult to defend these costs, especially when they are imposed on extremely poor communities where local inhabitants rely on the fish they catch as their only source of protein [28,29], unless there are unequivocal gains in terms of enhanced fisheries or other forms of poverty alleviation.

Inside MRs, full protection from fishing usually leads to a rapid increase in density and biomass of previously exploited populations. Species richness increases, alongside the size of individuals, and the age structure of fish populations [5,7,19,30]. Dividing species into targeted and non-targeted reveals that only target species tend to increase significantly in number within MRs; non-target species tend to remain the same or even decrease [20,30,31], due to an increase in predators inside the MR. MRs foster habitat recovery from fishing disturbances and allow different assemblages of species and habitat improvement (for example increased coral cover) [32]. In the Caribbean, MRs have been shown to enhance the recovery of coral reefs [33,34] by preventing the overfishing of herbivorous fishes that keep the substrate free for new coral recruits.

MPAs can generate conflicts between users (e.g. fishermen vs. others) and between objectives (e.g. conservation vs. fisheries). Opponents contend that many MPAs are just “paper parks”, impossible to properly enforce, or that they simply displace fishing effort to zones without effective management [35]. Others insist that while efforts to increase the size and number of MPAs must continue, solutions that stabilize the size of human population and our demands on biodiversity need to be found and implemented [36]. One of the main benefits proclaimed is the enhancement of fisheries around the areas of protection: they increase fish abundance inside the protected area and eventually this effect extends outside. However, whether MPAs truly enhance fisheries remains controversial [8,18,19,20,21,22,23,24].

Fisheries can profit from two different processes after the initial recovery inside the MR: the export of propagules (recruitment effect), and the export of adults (spillover effect) outside of the MR [37,38,39,40]. Additionally, fish from MRs are relatively naïve to fishing and therefore more easily fished [41]. The intensity of these effects, both inside and outside the MR, depends on: 1. Location, MRs with similar habitats inside and outside the border maximize spillover [42,43]; 2. Size, bigger reserves are preferable for conservation effects, but smaller MRs increase the border/area ratio and hence the spillover [42]; 3. Duration of the protection, abundances build up first inside the MR before spillover starts to happen [44]; 4. Isolation, whether the MR is isolated or part of a network (where the appropriate spacing between MRs is crucial); 5. Connectivity, exchange of larvae between the protected areas is especially important for conservation efforts [45] [46]. All these variables mean that the benefits of MRs are not always immediately detectable.

The dimensions and “visibility” of the spillover effect are critical to the acceptance of MRs by fishermen [5]. However, the effects of MRs can take from as little as 3 years [47,48] to several decades to be detectable [47]. Spillover can take even longer to detect, and the length of this period also varies among studies [6,20,40]. The spillover effect can differ from one taxonomic group to another and it also depends on whether the species is targeted by fishermen outside the MR [32]. In general when fishing intensity is low, the difference between the MR and outside areas is not significant [43]. Depending on all these factors, the spillover effect can be traced to 200–300m [6,49] or even 500m to 1–2km [20] from the MR border. Spillover can either be masked or reinforced by habitat variables [39] or by the amount of exploitation suffered before the MR was implemented [50]. Additionally, lack of compliance and enforcement can render MRs inefficient [35,51,52]. Also, high fishing intensity just outside the MR border, known as “fishing the line” [53], can mask the spillover effect, and increase the relative differences in fish abundance between the MR and the unprotected surrounding areas. Occasionally, the existence of an MPA attracts fishermen from other regions increasing fishing effort around it and contributing to the masking of the spillover effect [35]. This ‘attraction’ phenomenon is common in community-managed areas where legal ownership is hard to establish [54].

Spillover is the key to an MR being accepted as a fisheries-management tool. The strength and visibility of spillover effects are the main criteria by which local communities assess the success of an MR. This study is dedicated to the detection of these effects from a small, community-managed MR. Our main questions are: does the MR affect fish abundances? Can we detect spillover? Are there differences in spillover between trophic groups or fishes of different sizes? In a very remote and poor area such as the location of our study, Vamizi Island, Mozambique, the information gathered is crucial to allow Mozambican Government representatives and co-management institutions to assess the effectiveness of small community marine reserves in enhancing and supporting nearby fisheries [55,56].

## Material and Methods

### 2.1 Ethics

This study did not require animal ethics approval because no animals were handled. The field study did not involve endangered species. The study was carried out in Northern Mozambique (GPS coordinates: S 11°01,218'; E040° 40,762'). The Mozambican Government does not request or issue research permits to conduct research at this site. The study was authorized by the community committee in charge of managing this community reserve, as well as by the tourist resort operating in the area.

### 2.2. Study site

**Fig 1:**
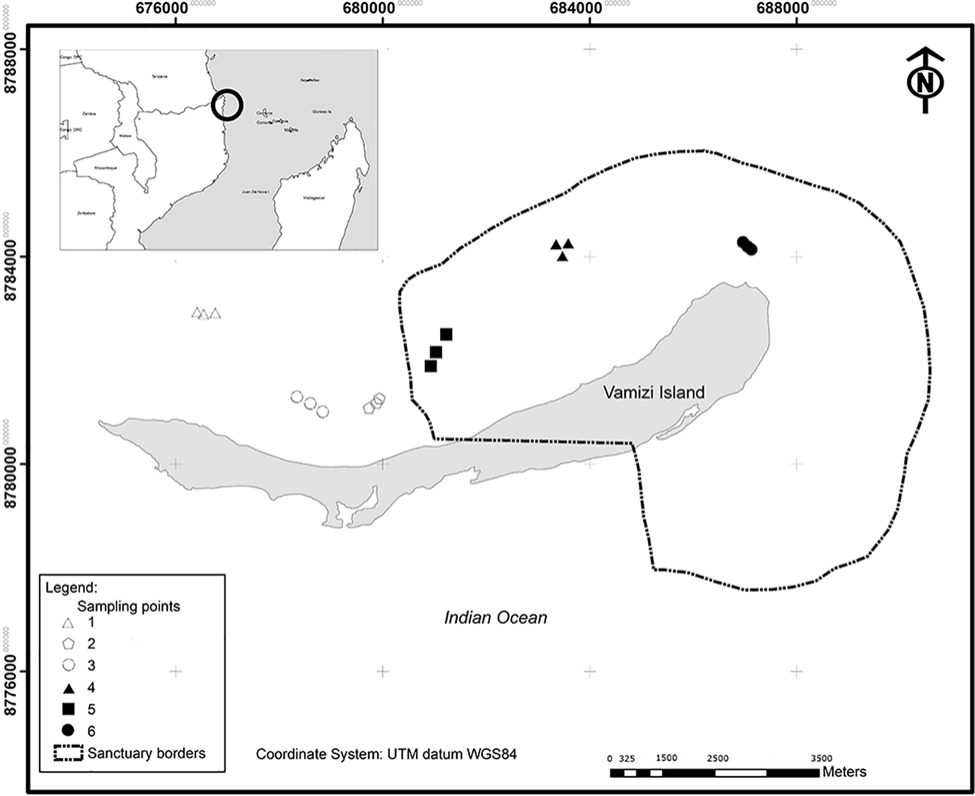
Map of Vamizi Island and location in Mozambique. Locations 1, 2 and 3 outside the MR. Locations 4, 5 and 6 inside the MR.

Vamizi is a 48km^2^ island located in the Quirimbas archipelago in Northern Mozambique (Fig 1), 40 km south of the Tanzanian border. The island has a permanent population of around 1500 people, which doubles in the dry season due to the annual migration of fishermen from Nacala, located in the adjacent southern province [57]. The island is far from urban centers and the region is largely undeveloped. On the east side of the island, an ecotourism lodge has been promoting conservation since 2000. Between December and March the predominant winds are from the north; the rest of the year they come from the south. To the north, Vamizi’s lagoon finishes in a reef crest, leading to a wall down to 200–500 m in eastern locations, and a slope to the same depths in the west. The south side of the island has a gentle slope into shallow reef flats of seagrass, macroalgae and coral bommies. On the north side, coral forms a continuous barrier with live cover between 30–60% and dominated by *Acropora* species [58].

In 2006, a 38 Km^2^ marine reserve was created by the community around the east point of the island, within which fishing is not permitted. The west point of the island around the villages was excluded from the marine reserve. The community enforces the no-take status with support from the lodge. In 2011, WWF (The World Wide Fund for Nature) began running a conservation project for the lodge, promoting the engagement of both the lodge and the communities with the marine reserve.

### 2.3. Sampling design

To quantify the effect of the MR, the state of reef communities was assessed using an Underwater Visual Census (UVC) at sites inside and outside the MR (Fig 1). Sites with similar wind, current and topographic characteristics were selected to facilitate comparisons between treatment (inside the MR) and control (outside the MR) locations. Surveys were conducted at two sites in 2003 (before MR establishment), at four sites in 2006 (during establishment) and at six sites in 2012 (6 years after establishment). In 2012 more sites were surveyed near the border of the MR to enable quantification of the spatial extent of the spillover effect (Fig 1). UVC is an indirect way of assessing spillover, as it does not involve tagging fish or tracking fish provenance. However, it is a reliable, non-destructive method with an established track record for detecting effects of MRs [38,39]. All of the 2012 surveys were conducted at 10m depth because surveys in 2003 and 2006 had shown no significant differences between two initial survey depths. Fish abundance data was collected in all years, but in 2006 and 2012 benthic variables (see below) were also collected.

At each site we quantified fish abundance, benthic cover and rugosity. Fish abundance is predicted to change directly as a result of the establishment of the MR. Quantifying benthic habitat and rugosity is important in order to control the effect of benthic variables on fish abundances allowing us to disentangle the effects of the MR and habitat factors. The positions of the survey sites along the border allow tracking of the changes in fish abundances along the gradient of the MR, and detection of spillover. UVCs [59] of reef fish were conducted to estimate abundances per 250m^2^ of herbivorous fishes families Acanthuridae and Scaridae, and of piscivorous families Lutjanidae, Haemulidae, and Serranidae (only groupers). Acanthuridae and Scaridae were chosen because of their ecological importance [60]. The piscivorous families were chosen as the most important indicators of overfishing [61]. Fishes were identified to genus and counted along 50m by 5m transects by following a transect line following a contour. Fish were counted within 2.5m on either side of the line. Each site had a nested design with 3 replicates and each replicate consisted of 3 transects, adding up to a total number of 18 transects in 2003, 36 in 2006 and 54 in 2012. To ensure standardization of transect width, 2.5m of tape was shown to the divers at the beginning of each survey dive. Some of the techniques of Samoilys and Carlos [62] were followed to count the fishes: the larger mobile fishes were counted first, then the smaller ones, and fishes that re-entered the transect area were not counted. In 2012 we also estimated the size of all the fishes surveyed by assigning them to size categories. At the beginning of each dive the observer was shown lengths of 10, 20, 30, 40 and 50 cm, at a distance of 2.5 m [62]. Additionally, the recording slate had size categories marked. The 10cm size categories are easy to use [63], and comply with methods commonly used in the Western Indian Ocean [64]. Length was converted to biomass using length-weight relationships in Fishbase [65]. Small numbers in each size category of piscivorous fishes prevented analyses of those data. Abundances were pooled into two functional groups for analysis: Piscivorous vs. Herbivorous.

Benthic cover was quantified following approaches commonly used in the Western Indian Ocean, to facilitate regional comparisons [60]. Specifically, we used photo transects consisting of photos taken in 20m transects with 2 photos per meter, one on each side of the transect (i.e. 40 photos per transect) to avoid pseudoreplication. The sampling design was similar to that previously described for the fishes: each site had 3 replicates, each replicate 3 transects, and each transect 40 photos. Photos were analyzed with CPCe 3.2, Coral Point Count w/ excel extension from NCRI. This software gives the mean cover for several benthos categories based on 10 random points for each photo. Each random point is inspected and classified as hard coral, soft coral, dead coral with algae, recent dead coral, macroalgae, coralline algae, other invertebrates, and sand, rubble or pavement. Rugosity was estimated by measuring the contour of the reef under a portion of 5m of the transect line, the value was then divided by 5m, and used as index of reef complexity [66] and was only measured in 2012. Benthic cover estimates and rugosity estimates, allow controlling for differences in complexity among sites, and hence identifying situations where the faunistic differences between the locations are caused by differences in the habitat.

### 2.4. Data analysis

Data were categorized as benthic cover variables, fish abundance variables (numerical abundance and biomass for herbivorous fish), or time /spatial traits (MR years/distance to the border). Fish abundance was the response variable and all the other variables were assessed as predictors of fish abundance.

Regression trees were used to identify the most important predictor variables. Regression trees consist of a series of binary splits of the response variable based on the values of the predictor variables (we did not transform the predictor variables). They are constructed by recursively partitioning the data set of fish abundances into two subsets based on the optimal split among all possible splits, where optimality is defined as the reduction of the mean squared error. Since we cross-validate the results (10 fold), the optimal tree is the one with the smallest Cross Validated Relative Error (CVRE) or the smallest size plus one standard deviation [67]. The output is a tree diagram with the branches determined by the splitting rules based on the predicator variables: MR years, distance to the border, benthic cover variables and rugosity. Regression trees identify differences in fish abundance and the location of changing points for these differences. Two regression trees were constructed splitting fish abundance (herbivorous and piscivorous) by all the benthic variables, distance and years. Using the results of the regression tree, we built several Generalized Linear Models (GLMs) models with the abundance of fish as a function of year and distance to the reserve boundary with appropriate breakpoints for each year (Tables 1 and 2).

**Table 1:** Summary data of the multivariate regression tree constructed based on herbivorous fish abundances.

Regression tree	CVRE	Number Splitting nodes	1° Node	2°Node	3° Node	4ªNode
Abundances of herbivorous fishes	0,342	3	Y = 9	D = -1,55	D = -0,95	
*All variables						
Abundances of piscivorous fishes *	0,0833	1	D = 4			
Distance * Years						

We present the values for: the CVRE (Cross Validated Relative Error), the number of splitting nodes and variable prediction values for each node. D-distance, Y-years.

**Table 2:** AIC (Akaike information criterion) values for GLMs with different breakpoints.

	AIC
**herbivorous fishes**	
**2006**	
model without breakpoint	297,3*
model with breakpoint of dis = -1,55	297,59
model with breakpoint of dis = -0,5	299,51
model with breakpoint of dis = 3,6	299,28
**2012**	
model without breakpoint	972,23
model with breakpoint of dis = -1,55	712,86*
model with breakpoint of dis = -0,5	933,07
model with breakpoint of dis = 3,6	917,33
**Piscivorous fishes**	
**2006**	
model without breakpoint	418,08
model with breakpoint of dis = -1,55	345,76
model with breakpoint of dis = -0,5	322,81*
model with breakpoint of dis = 4	327,66
**2012**	
model without breakpoint	1471,4
model with breakpoint of dis = -1,55	1446,7
model with breakpoint of dis = -0,5	1456,6
model with breakpoint of dis = 4	1359,5*

The model with lowest AIC is chosen *. Dis- Distance.

Assuming a Poisson distribution for the fish abundance, *Z*_*y*_, of year *y*, we fit a Generalized Linear Model (GLM) with a break point. Each GLM models how the mean abundance *E*⎣*Z*_*y*_⎦ of each year changes according to the distance, x, to the reserve boundary:

$$E\left[Z_{y}\right]=\exp[\beta_{0}+\beta_{1}xI\left(x\leq x^{*}\right)+\left\{\beta_{2}+\beta_{3}\left(x-x^{*}\right)\right\}I\left(x>x^{*}\right)],$$

where x* is the breakpoint and *I*(*A*) is an indicator function taking 1 when *A* is true, or 0 otherwise. This non-linear model is therefore discontinuous at the change point x*.

The best model for the year and trophic group was chosen using AIC (Akaike information criterion). The total number of individuals per 250 m^2^ transect of each of the two functional groups was modeled as a Poisson random variable. All the model parameter estimates used to construct the graphic representation are presented with their standard error (Table 3).

**Table 3:** β Value estimates and respective standard error for the GLM models.

Herbivorous model					
Year	*β*_0_ (std. error)	*β*_1_ (std. error)	*β*_*2*_ (std. error)	*β*_3_ (std. error)	x*
2003	3,272 (0,046)	0,004 (0,012)	-	-	-
2006	3,478 (0,031)	-0,037 (0,007)	-	-	-
2012	3,323 (0,167)	-0,363 (0,046)	1,986 (0,170)	0,003 (0,006)	-1,55
**Piscivorous model**					
**Year**	*β*_0_ **(std. error)**	*β*_1_ **(std. error)**	*β*_2_ **(std. error)**	*β*_*3*_ **(std. error)**	X*
2003	1,505 (0,111)	0,003 (0,029)	-	-	-
2006	-0,213 (0,381)	-0,426 (0,105)	-3,268 (0,771)	0,898 (0,091)	-0,5
2012	1,403 (0,088)	0,431 (0,030)	2,336 (0,128)	-0,079 (0,035)	4,0

The benthic cover data were analyzed with Nonmetric multidimensional scaling (NMDS) to evaluate the homogeneity of the habitat between the inside and outside of the marine reserve. NMDS was used, since the data are proportional and thus non-independent, and zeros were frequent. NMDS represents the set of objects along a predetermined number of axes while preserving the ordering relationships among them. We used Gower distances and expanded scores based on the Wisconsin square root of the data. The goal of this analysis is to investigate if the benthic characteristics of the sites vary consistently as a function of the distance to the reserve boundary and/or time since reserve implementation. As such, it provides an additional exploratory indication of the effects of the habitat as potential driver of fish abundances. All the data are presented in S1 Table. All analyses were carried out in R [68] with the following packages: vegan for the NMDS [69], mvpart for regression trees [70], and car for the GLMs [71].

## Results

**Fig 2:**
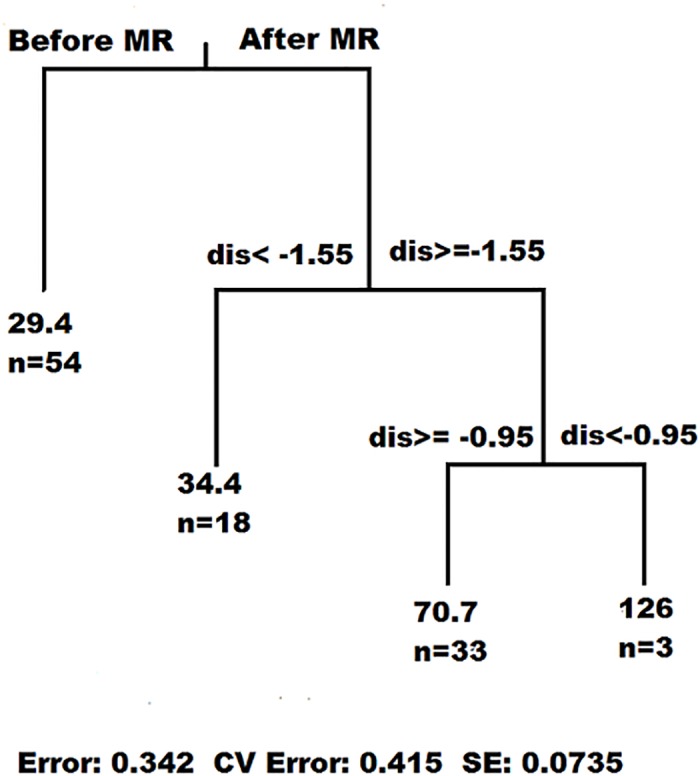
A multivariate regression tree was built based on herbivorous fish abundances. For each node the mid-point value of the split is reported, and on each leaf the number of observations (n) on that leaf.

In the herbivorous fish abundance tree, four branches appeared, the first node indicating that in 2009 (the mid-year between 2006–2012), herbivorous fish abundance started to increase, followed by nodes at distances of -1,55 and -0,95km (outside the reserve). Herbivorous fish abundance substantially increased towards the MR (Fig 2). The points -1,55 and -0,95km are the mid-points between sampling sites where a break occurs. None of the benthic variables appeared in the nodes of the trees (Table 1).

The regression tree analysis for the piscivorous fish abundance produced a tree with only 2 branches and one node (Table 1), dividing abundances of fishes at 4km distance from the border, well inside the MR boundaries, while the variable years did not appear in any node, nor did any of the benthic variables. The abundances of piscivorous fishes are only very different well inside the border of the MR.

**Fig 3:**
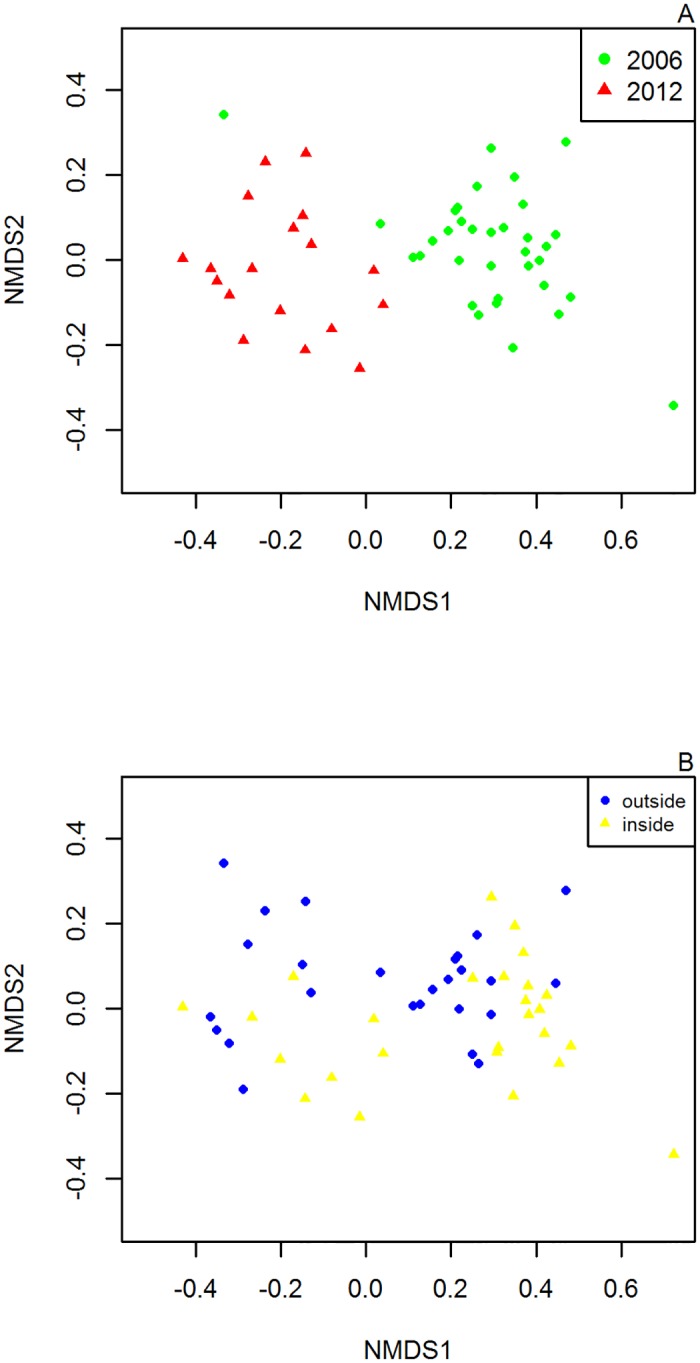
Nonmetric Multi Dimensional Scaling (NMDS) of the benthic observations by: A- Year of observations B- Distances to the border. (negative values outside the border, positive values inside the border)

To consolidate the results of the regression tree, we compared sites according to benthic cover variables using NMDS (Fig 3). No aggregation by distance from the MR boundary (Fig 3A) is apparent from the ordination, but different years appear to segregate with regards to their benthic cover (Fig 3B). NMDS stress for this model is 0,139.

**Fig 4:**
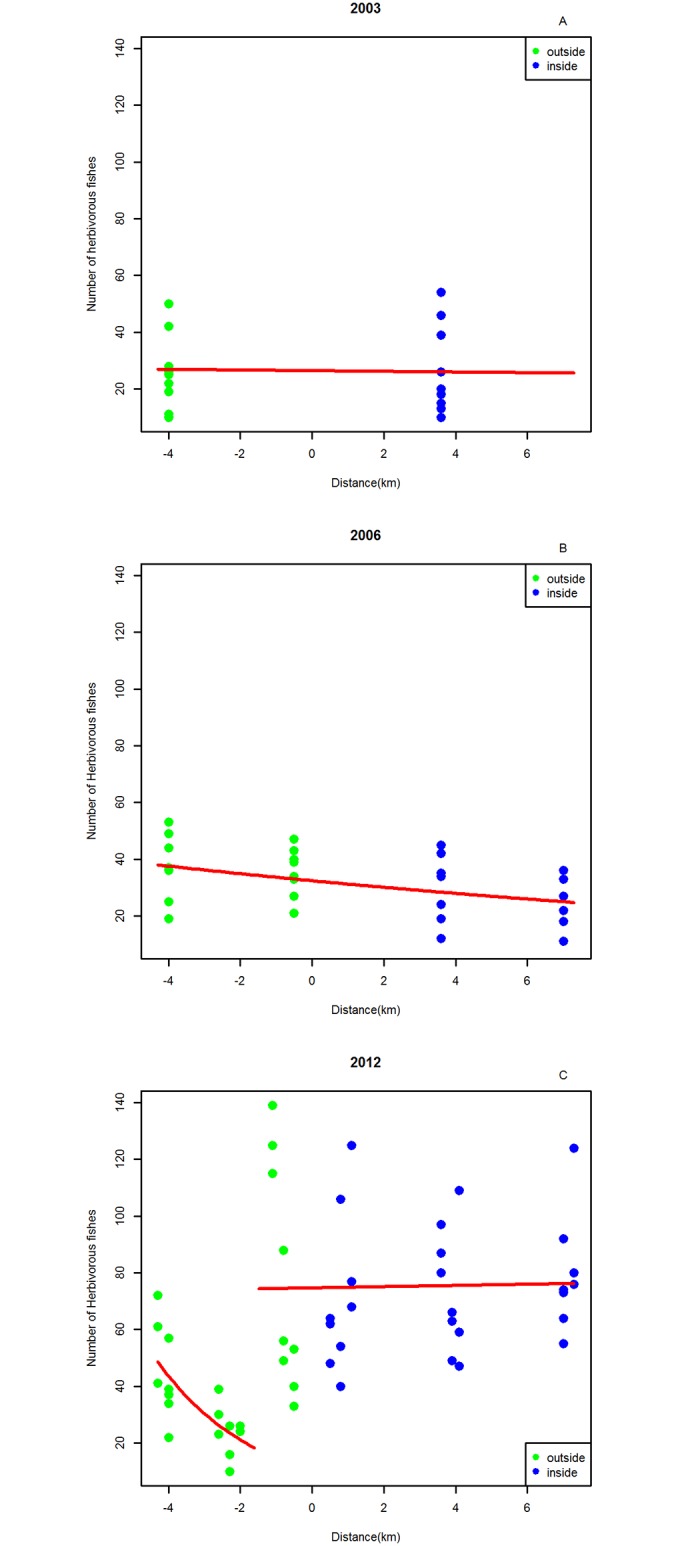
Summary of Generalized Linear Models (GLM) for herbivorous fishes abundance. Points with negative distance are situated outside the MR and points with positive distance are situated inside the MR. The red line reflects model predictions. A- 2003. B- 2006. C- 2012.

We used the results from the regression tree to build three GLM models of fish abundance for each year as a function of distance to the MR border. The models had either no breakpoint, or a breakpoint outside the reserve at 1.55 and 0.5km from the border (dis = -1.55 and −0.5), and inside the reserve at 4km (dis = 4) from the border (Table 2). Fig 4 shows that for herbivorous fish abundance only in 2012 did model selection favor a model with a breakpoint, with the break point at -1.55km (Fig 4C). The 2006 model with lowest AIC did not have any break points (Fig 4A and 4B), and no breakpoints were apparent in 2003 either.

**Fig 5:**
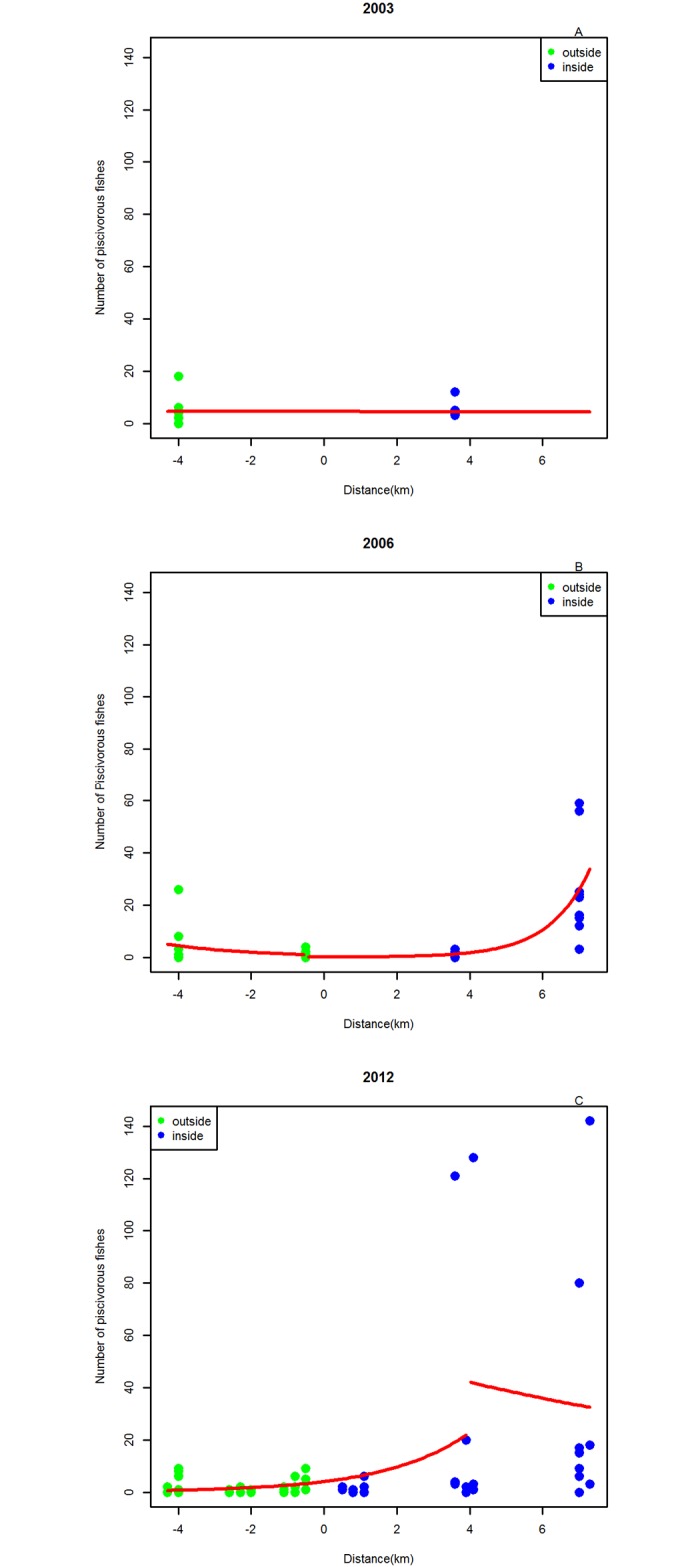
Summary of Generalized linear Models (GLM) for piscivorous fish abundances. Points with negative distance are outside the MR and points with positive distance are inside the MR. The red line reflects model predictions. A- 2003. B- 2006. C- 2012.

For the piscivorous fish, GLM models show that 2003 data also have no distinguishable breakpoint (Fig 5A), while for 2006, the models with a break point at -0,5 and 4km from the border did have very similar (lower than no breakpoint, but not a big difference) AICs (Table 2) and the lower AIC model was represented (Fig 5B). Finally, in 2012 the model that best fitted the data was the one using the splitting node of the Multivariate Regression Tree, located at 4km inside the reserve (Fig 5C). All the parameter estimates of the models used are given in Table 3 with their standard error.

**Fig 6:**
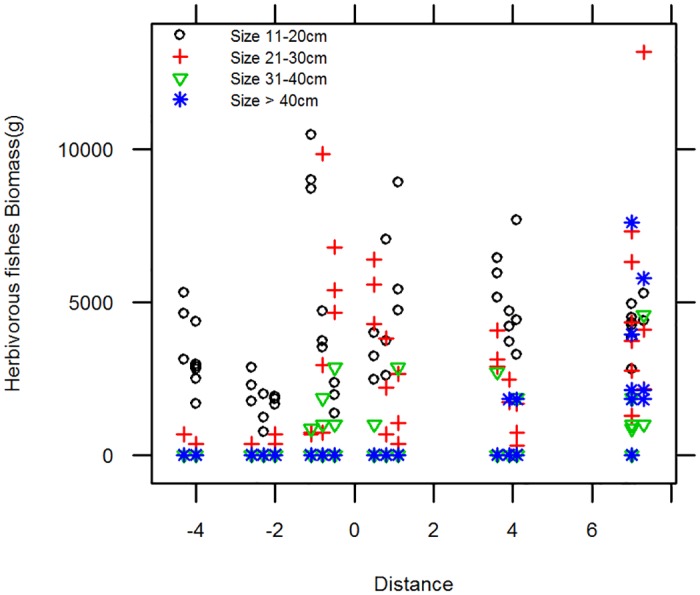
Scatter plot of the biomass of herbivorous fish by size. Negative distances are outside the MR, positive distances are inside and 0 is the border of the MR.

The biomass of herbivorous fishes peaked at the border zone (Fig 6) and inside the MR. The smallest sizes classes are responsible for the higher biomass at the MR border (for sizes 10–20cm and 21–30cm). Large herbivorous fish only occur well within the reserve boundaries.

## Discussion

Our data showed the existence of a significant and consistent effect in all trophic groups on the abundance and distribution of reef fishes of the Vamizi marine reserve. Based on the regression tree splits, six years after its establishment, both herbivorous and piscivorous fish are more abundant inside the reserve than outside, where no difference existed before. Additionally, spillover was detected in the herbivorous fish functional group, but not in the piscivorous group, which is only more abundant well within the reserve. Most importantly, fish abundance outside the reserve has not decreased in relation to its abundance prior to the reserve establishment, despite the concentration of fishing pressure.

Our Before-After-Control-Impact sampling design and the analysis of benthic variables confirm that habitat differences were not responsible for the variation in fish abundances. Based on the regression trees results our analysis relies on the assumption that the habitat is uniform, so that the differences in the abundance of fish can be attributed to the reserve establishment. However, protection could have changed the habitat and influenced reef fish abundances since MR establishment. For that reason we included benthic variables in the study. There is conflicting evidence in previous studies: in some, habitat accounts for part of the variation [31,39,72], in others habitat had no effect [49]. These apparent differences are likely caused by the specific characteristics of each study site. Nevertheless, McClanahan [73] stated that reef structure has less influence on reef fish abundances than management, and our findings agree with this. Our NMDS plots are in accordance with the results from regression trees, showing apparent differences between the years (due to management) but not between different distances (due to habitat differences). Moreover, evidence suggests that habitat homogeneity around the reserve enhances spillover, increasing the distance from the border at which it is detectable.

Recovery from fishing is often different for piscivorous and herbivorous functional groups. Recovery will also depend on which fish are targeted by fishermen [49]. In Vamizi island both piscivorous (especially groupers) and herbivorous fish (especially parrotfish) are targeted by fishermen. Results from other studies found that recovery of predatory fishes was the largest effect of marine reserve establishment, while the response by herbivorous fish was weak [74]. In some cases, spillover was detected for predatory species only [75], while others found that the predators have a slower response and that they eventually reduce their herbivorous prey [30,76,77]. McClanahan et al. [78] found that different groups react differently: Scaridae and Labridae increase rapidly, Balistidae and Acanthuridae slower, and some predators may never recover. The results vary between studies, and few are focused on spillover, the majority of studies tending to concentrate on fish recovery inside the reserve. We predict that, with time, the spillover effect for herbivorous fish will change relative to the extent of piscivorore spillover. Similar changes were revealed for the recovery of fishes inside MRs by other authors in analogous studies [60].

Herbivorous fish size results indicate that smaller-sized fishes are responsible for the spillover effect in 2012. We suggest that an increase in predators and agonistic relations within the protected area drove the smaller individuals out of the reserve, a pattern already detected in other studies [49]. This interpretation would reconcile our findings- more small fishes on the border of the reserve- with the literature, namely regarding the higher number of larger fishes and predators inside MRs [43,79]. Another explanation could be that the larger Scaridae are too big to be eaten by the predators, meaning that the smaller fish within the reserve are predated upon more heavily and consequently their numbers are lower. An alternative possibility is that herbivorous fishes are the first to recover and hence the first to be detected in terms of spillover effects. This is supported by McClanahan and Mangi [20] who point out that Scaridae (one of the most abundant herbivorous groups) are the fastest recovering group inside reserves. Herbivorous fishes are of great importance to reef health maintenance, keeping algae from competing for space with corals. Good numbers of herbivores are a sign of reef resilience to climate change [60]. In the context of MPAs, protection and recovery of herbivorous fishes is of major importance to the conservation and recovery of coral cover and health [33,79].

Our study reports a larger area of spillover than most other studies. We found a spillover distance of more than 1km outside of the reserve, which contrasts with the distance values of 300–350m reported by some authors [38,49], or the 500m reported by others [80] [20,43]. One of the reasons for this larger spatial extent of spillover could be the homogeneity of the habitat around the border of the MR, a characteristic previously highlighted as a multiplier factor of the reserve’s effects [81]. In the presence of low fishing pressure, a 1.2km spillover distance has been occasionally reported [20,43]. Nonetheless, other studies that found a very light spillover effect and only for a few meters, attributed the weak spillover to “fishing the line” [53,80]. However, even observing heavy “fishing the line” around Vamizi MR, the spillover effect could still be detected.

The regression tree results reveal changes in fish abundances since the designation of the Vamizi reserve (less than 6 years). These findings are consistent with the literature which reports time intervals from establishment to detection from as little as 3 years [61,82,83], although in some cases it has taken decades before recovery is detected [30]. This recovery could be explained by the initial state of the Vamizi MR, which was not severely depleted because only light fishing (involving fewer than 120 fishermen) was had occurred, or by the strong compliance to the marine reserve restrictions. Our 2003 data, from before the MR establishment, support the former explanation [58]. Fishing pressure was light outside the reserve (stronger on the border of the MR) in the sense that fisheries around Vamizi are mainly artisanal and subsistence fisheries, not using trawling or mosquito nets as in mainland Mozambique. Meanwhile, fishermen numbers did increase in Vamizi from the original 120 from 2003, to approximately 131 in 2006, and 159 in 2012, but this does not necessarily imply a large increase in fishing pressure, because the increase was mainly in speargun and line fishing, just for few days a week and only 0–20kg a day, as opposed to the 20–180kg a day by those using gillnets and seine nets whose number did not increase in this area. Moreover, compliance with the marine reserve rules was variable (but better than most MRs) [44] and also, only migrant fishermen tried to fish in the MR. This is confirmed by another study in which Vamizi MR was classified as having variable compliance levels [84].

Most of the controversy surrounding MPAs concerns the benefits to fisheries. MPAs have been showing improved fish abundances inside the protected areas, but less is known about the spillover effect, which was the main focus of our work. We show that habitat homogeneity is important for spillover, that spillover can be achieved in 6 years and that it is different across the different trophic groups. Vamizi marine reserve has variable compliance levels and reasonable enforcement of fisheries laws, and spillover was still detected. We suggest that small community marine reserves must be well placed (with homogenous habitat around the border), should have good enforcement and the fishing activities around the MR need to be well managed (controlling fishing effort and fishermen number), to achieve best performance in terms of enhancing the fisheries through spillover.

## Supporting Information

S1 Table Raw data table. (CSV)
